# CircRNA mmu_circ_0000021 regulates microvascular function via the miR-143-3p/NPY axis and intracellular calcium following ischemia/reperfusion injury

**DOI:** 10.1038/s41420-022-01108-z

**Published:** 2022-07-11

**Authors:** Jingjie Xiong, Yisen Hu, Yi Liu, Xiaocong Zeng

**Affiliations:** 1 Department of Cardiology, The First Affiliated Hospital of Guangxi Medical University, Nanning, Guangxi China; 2 Guangxi Key Laboratory Base of Precision Medicine in Cardio-cerebrovascular Diseases Control and Prevention & Guangxi Clinical Research Center for Cardio-cerebrovascular Diseases, Nanning, Guangxi China; 3 School of Basic Medical Sciences, Guangxi Medical University, Nanning, Guangxi China

**Keywords:** Vascular diseases, Mechanisms of disease

## Abstract

Cardiac ischemia-reperfusion (I/R) is associated with a high rate of complications. Restoring microvascular function is crucial for cardiac repair. However, the molecular mechanisms by which the circRNAs repairs microvascular dysfunction are unknown. High-throughput RNA sequencing and quantitative real-time PCR (qRT-PCR) were used to measures circRNA levels in cardiac tissue samples. We found a total of 80 up-regulated and 54 down-regulated differentially expressed circRNAs, of which mmu_circ_0000021 were consistent with bioinformatics predictions. Next, mmu_circ_0000021 knockdown and overexpression were performed to indicate the functional role of mmu_circ_0000021. The interaction of mmu_circ_0000021, miR-143-3p and NPY were evaluated using dual-luciferase assays, RNA pull-down assays and RNA immunoprecipitation (RIP). Immunohistochemistry, transmission electron microscopy, and immunofluorescence were used to determine the presence of leukocytes and changes in microvascular morphology and function. Mechanistically, mmu_circ_0000021 involved in regulating microvascular dysfunction via miR-143-3p by targeting NPY. However, the contraction of microvascular spasm caused by NPY is related to calmodulin. By regulating NPY, Circular RNA (circRNA) further affects microvascular spasm, regulates microcirculation disorders, and restores cardiac function. Our findings highlight a novel role for mmu_circ_0000021 by regulating microvascular function following I/R injury.

## Introduction

Active reperfusion therapy for individuals with acute coronary syndrome includes thrombolysis or primary percutaneous coronary intervention, both of which improve the long-term prognosis of ST-segment elevation myocardial infarction patients [1]. Unfortunately, while reperfusion treatment restores blood flow, microvascular reperfusion damage often occurs. This situation is referred to as “no-reflow” and is characterized by cardiac tissue hypoperfusion [2]. Upon reperfusion, substantial endothelial cell swelling, microvessel wall rupture, and bleeding into the interstitial space are common signs of microvascular damage [3]. Calcium overload can lead to endothelial damage and microvascular injury. During cardiac I/R, increased cytosolic calcium induces calcium entry into the mitochondria through the mitochondrial calcium uniporter, activating the mitochondrial permeability transition pore, and causing cell apoptosis [4]. Leucocyte adherence and microembolization, which generate microvascular endothelial hyperpermeability and junctional loss, may cause microvascular dysfunction [5]. Importantly, this happens despite the restoration of normal epicardial flow, further aggravating cardiac injury [6]. Since cardiac microvascular dysfunction is the result of no-reflow to the cardiac tissue, targeted molecular therapy of the microvasculature may significantly decrease pathologic remodeling and improve outcomes [7, 8].

Neuropeptide Y (NPY) is a member of the G protein-coupled receptor superfamily that has been shown to increase both cytosolic and nuclear calcium levels [9]. Plasma NPY levels were linked to reperfusion and the coronary microvascular resistance index in a recent study. NPY can also trigger excitation-contraction coupling between cardiomyocytes and vascular endothelial cells by acting on Y1 receptors, which increases intracellular calcium ion concentrations and subsequent cardiomyocyte apoptosis and microvascular endothelial dysfunction [10, 11]. Furthermore, Previous researches indicated that reduced SR Ca^2+^ load and Ca^2+^ transient amplitude were responsible for cardiac I/R [12]. The release of calcium by the sarcoplasmic reticulum through the sarcoplasmic/endoplasmic reticulum calcium ATPse 2a (SERCA2a) or ryanodine receptor 2 (RyR2) is crucial for cardiac inotropy [13]. Cardioprotection can be achieved by suppressing circulating NPY levels and affecting NPY-NPY1R signaling [14]. Cardiomyocyte survival and mitochondrial membrane potential are negatively impacted by NPY overexpression, which is mediated by calcineurin (CaN), calcium AMP kinase II (CAMKII), and p38 signaling pathways [15]. These findings indicate that NPY may exacerbate myocardial microcirculation disturbance by regulating calcium overload-related mechanisms to promote cardiomyocyte apoptosis [16, 17]. However, the molecular mechanism associated with NPY and circRNAs to improve microvascular function is still unclear.

Circular RNAs (circRNAs) are a complicated category of noncoding RNAs characterized by a covalently closed loop structure that is only expressed during specific developmental stages [18, 19]. It has been demonstrated that circRNAs play a significant role in a number of clinical disorders, including cardiac I/R and gene regulation [20, 21]. For example, circPVT1 inhibits miR-125b and miR-200a-mediated apoptosis signal transduction to protect coronary blood vessels from damage [22]. MicroRNAs (miRNAs) are known to act as molecular sponges that bind to target mRNAs and negatively impact the synthesis of target mRNAs as a result of their association with circrRNAs [23]. While multiple miRNAs have been implicated in modulating vascular function, the particular miRNA involved in cardiovascular healing are rarely reported [24].

In this study, we performed a high throughput sequencing to detect circRNAs expression variations. The interactions between circRNAs and miRNAs were predicted by prediction software. Mmu_circ_0000021 was validated as the target gene by sequencing data and mir-143-3p was the most linked with it. Mmu_circ_0000021 was shown to be elevated in vivo and in vitro models by stimulating calcium influx and cell apoptosis. Inhibition of mmu_circ_0000021 expression also ameliorated microvascular abnormalities disorders. Therefore, mmu_circ_0000021 may play a role in microcirculation development by binding competitively to miR-143-3p and regulating NPY expression. Our findings provide a new insight into preserving cardiac microcirculation and present a possible therapeutic strategy for no-reflow following cardiac I/R.

## Results

### Differentially expressed circRNAs

**Fig. 1:**
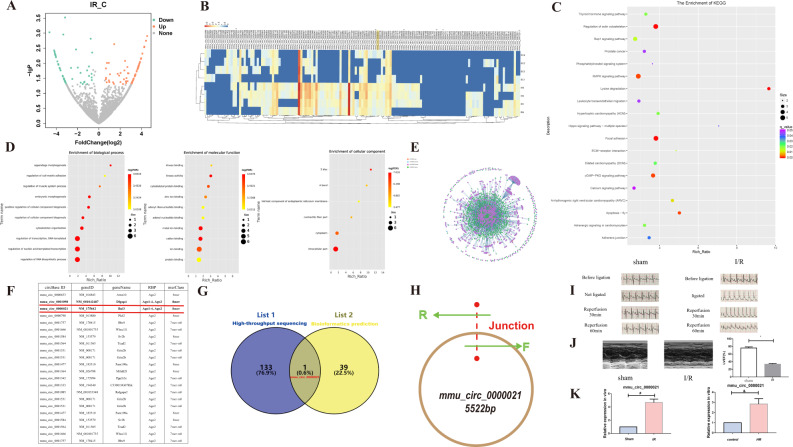
Screening for differentially expressed circRNAs. **A** Volcano map of up-regulated or down-regulated differentially expressed circRNAs. **B** Heatmap illustrating differentially expressed genes identified by high-throughput sequencing. **C**, **D** GO and KEGG analysis analyses were performed using Clusterprofiler. **E** Cytoscape software was used to visualize an interaction network based on miRNA and circuRNA interaction data. **F** Starbase shows the predicted circRNA and miR-143-3p. **G** The Venn diagram illustrates the common gene target between bioinformatics prediction and circRNA sequencing. **H** CircRNA that exhibits a ring structure. **I** ECG recordings were analyzed for different periods. **J** Echocardiography was used to evaluate cardiac function in mice. **K** Changes in mmu_circ_0000021 in vivo and in vitro models were tested by qRT-PCR. #*P* < 0.05 compared with the sham group; &*P* < 0.05 compared with the control group; *n* = 8/group.

The high throughout sequencing of 4 paired mice was used to investigate the differential expression of circRNAs between myocardial tissues (SC1, SC2, SC3, SC4) and cardiac tissues following I/R injury (IR1, IR2.IR3.IR4). The heatmap generated from the high-throughput sequencing results revealed 134 differentially expressed circRNAs, including 80 up-regulated and 54 down-regulated transcripts (Fig. 1A, B). Calcium signaling and apoptosis pathways were enriched in the KEGG pathway analysis (Fig. 1C). GO analysis showed that the most abundant biological processes (BPs) were regulation of RNA biosynthetic process, the most abundant molecular functions (MFs) were protein binding and the most relevant cellular components (CCs) were intracellular components (Fig. 1D). An interaction network based on miRNA and circRNA interaction data was generated using Cytoscape software and predicted of miR-143-3p-related circRNAs by bioinformatics (Fig. 1E, F) [25]. The intersection of the overlapping circRNAs between high-throughput sequencing and bioinformatics prediction data subsets is shown in a Venn diagram (Fig. 1G). Although mmu_circ_0000021 is in a class of noncoding RNAs with a closed loop structure formed by covalent bonds (Fig. 1H) [26], its function in myocardial I/R remains unknown. We preliminarily validated the established I/R model and the reliablity of transcriptome sequencing data was verified through qRT-PCR analysis. (Fig. 1I-K). Next, we investigated the function and potential mechanism of mmu_circ_0000021 following I/R.

### miR-143-3p is a downstream target of mmu_circ_0000021

**Fig. 2:**
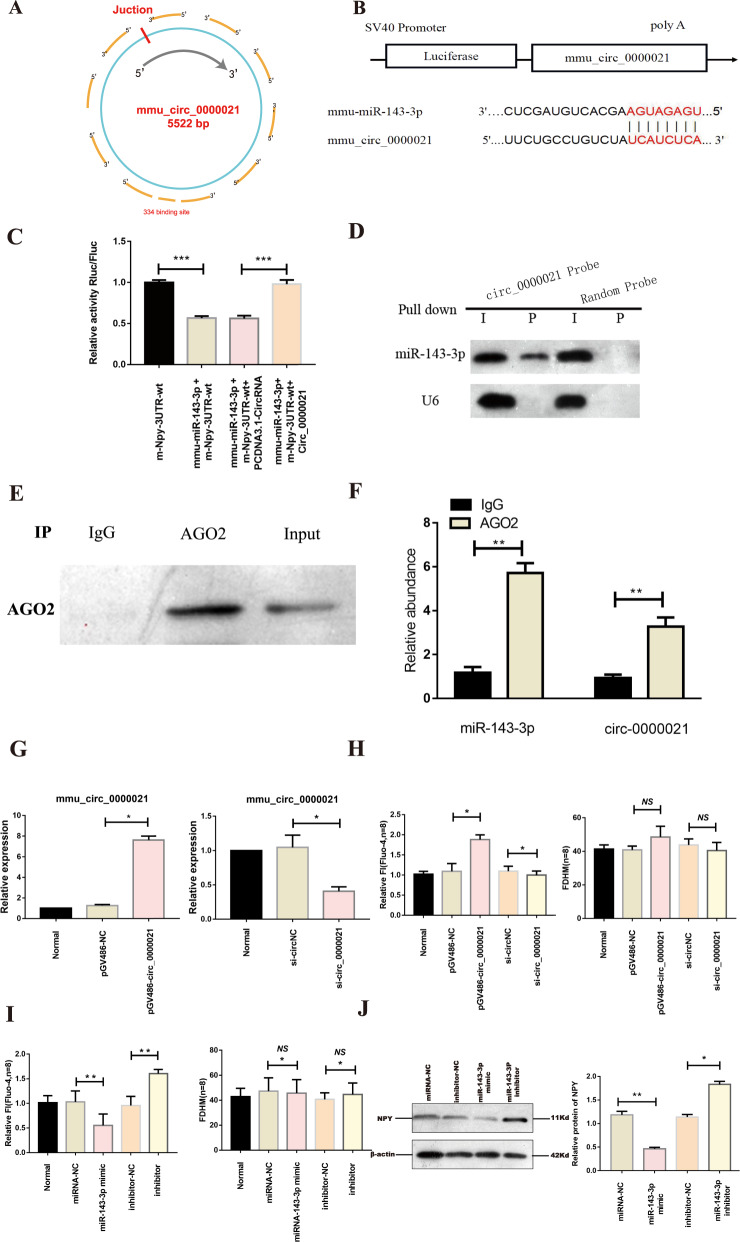
MiR-143-3p is the downstream target of Mmu_circ_0000021. **A** The 334 possible binding sites of miR-143-3p and mmu_circ_0000021. **B** The binding target of circRNA and miRNA. **C** PCDNA3.1-circ_0000021 staining reduced the fluorescence density of NMCMs labeled with miR-143-3p. **D** MiR-143-3p is pulled down by the circRNA probe or random probe. miR-143-3p levels are analysed by northern blot. **E**, **F** The RIP assay for circ-0000021 was performed with an anti-AGO2 antibody in NMCMs. **G** Left: pGV486-circ_0000021 can significantly increase the level of mmu_circ_0000021; Right: siRNA can significantly reduce the level of mmu_circ_0000021. **H** Fluo-4’s peak intensity increased following overexpression of mmu_circ_0000021, and vice versa; bar graph shows that up-regulation or down-regulation of mmu_circ_0000021 has no influence on FDHM. **I** The peak FI of fluo-4am reduced in NMCMs transfected with miR-143-3p, and the FI rose when the expression was suppressed. In contrast, the FDHM of NMCMs did not change in response to miR-143-3p or inhibitors. **J** Western blot analysis was used to measure NPY expression and ImageJ software was used to quantify relative proteins. *P < 0.05, **P < 0.01 vs. indicated group; NS not significant, *n* = 8/group.

We used miRanda 2010 and RNA hybrid-2.1.2 to identify the downstream target of mmu_circ_0000021, which has 334 potential binding sites (Fig. 2A). Further research was carried out to investigate the direct connection between mmu_circ_0000021 and miR-143-3p. The binding potential of the two RNAs was determined using a dual-luciferase reporter experiment. The results demonstrated that miR-143-3p inhibited the luciferase activities of the wild-type and mutated mmu_circ_0000021 reporters, but not the activity of the mutated mmu_circ_0000021 reporter with two mutated binding sites (Fig. 2B, C). Northern blotting revealed that the circ-0000021 probe could also pull down miR-143-3p in reverse (Fig. 2D). The binding of miR-143-3p to mmu_circ_0000021 was then investigated using an avidin-biotin pull down experiment. Argonaute 2 (Ago2) is an essential component of the RNA-induced silencing complex (RISC), in which miRNAs mute genes and are controlled by circRNAs. Our results showed that mmu_circ_0000021 and miR-143-3p were significantly enriched, as they were precipitated by the anti-AGO2 antibody (Fig. 2E, F). The above findings demonstrated that mmu_circ_0000021 could directly bind miR-143-3p in NMCMs. Overexpression of mmu_circ_0000021 increased cytoplasmic calcium levels, while knockdown had the opposite effect, as validated using calcium ion probes. However, changing mmu_circ_0000021 expression had no effect on the duration of the calcium transient (Fig. 2G, H). We found that suppressing or boosting miR-143-3p expression in vitro had the opposite effect (Fig. 2I). MiR-143-3p inhibited NPY in cardiomyocytes by Western blot analysis (Fig. 2J). The above data showed that miR-143-3p may function as a link between mmu_circ_0000021 and calcium in the heart. However, the underlying mechanism has yet to be determined.

### NPY is the target of Mmu_circ_0000021 in NMCMs

**Fig. 3:**
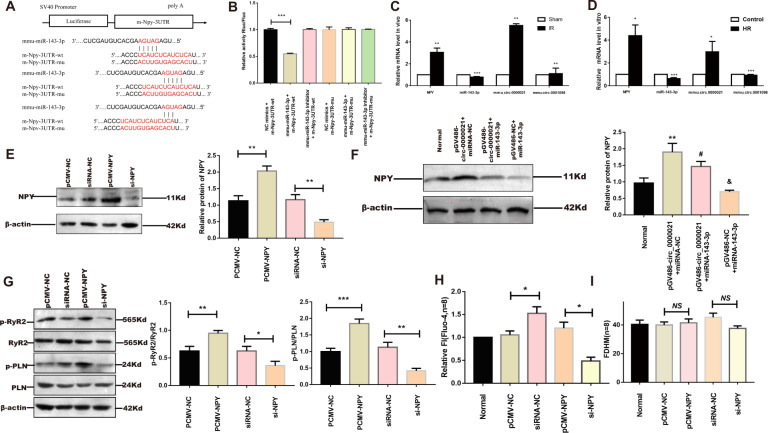
NPY is the target of mmu_circ_0000021 in NMCMs. **A** Target binding of miR-143-3p and NPY. **B** Dual-luciferase reporter assay for miR-143-3p and NPY. **C**, **D** In vivo and in vitro experimental verification of mmu_circ_0000021, expression of miR-143-3p and NPY. **E** Gene and protein expression of NPY after plasmid transfection. **F** Western blot showing that the increase of NPY caused by mmu_circ_0000021 was attenuated by miR-143-3p. **G** Overexpression of NPY increased the level of p-RyR2 and p-PLN and vice versa. **H** Overexpression of NPY increased the fluo-4am value in NMCMs. *P < 0.05; n = 8/group. **I** Bar graph showing that changes in NPY in NMCMs had little effect on Ca^2+^ transients. *P < 0.05, ***P < 0.001 vs. indicated group; NS not significant, *n* = 8/group.

Luciferase activity was significantly decreased in the miR-143-3p + NPY-3UTR-wt compared to the NC mimic + NPY-3UTR-wt (*P* < 0.05). Compared to the NC group, mmu-miR-143-3p had no effect on m-Npy-3UTR-MUT luciferase expression (P > 0.05), indicating that the mutation was effective (Fig. 3A, B). Gene expression was subsequently confirmed both in vivo and in vitro. mmu_circ_0000021 and NPY mRNA expression increased, whereas miR-143-3p expression decreased (Fig. 3C, D). Overexpression of NPY raised protein p-RyR2, p-PLN, and the peak concentration of Ca^2+^ in NMCMs, whereas a reduction in NPY decreased p-RyR2, p-PLN, and Ca^2+^ peak. And NPY change had minimal influence on the Ca^2+^ transient. (Fig. 3G-I) Protein NPY was increased in NMCMs transfected with pGV486-circ_0000021, and miR-143-3p mimic may inhibit this upregulation. (Fig. 3E-F) Through the mmu_circ_0000021 - miR-143-3p - NPY axis, mmu_circ_0000021 might impact the Ca^2+^ content in the cytoplasm, as indicated by the data presented above.

### MiR-143-3p is responsible for mmu_circ_0000021-mediated calcium influx in cardiomyocytes and alleviates H/R-induced cardiomyocyte apoptosis via targeting NPY

**Fig. 4:**
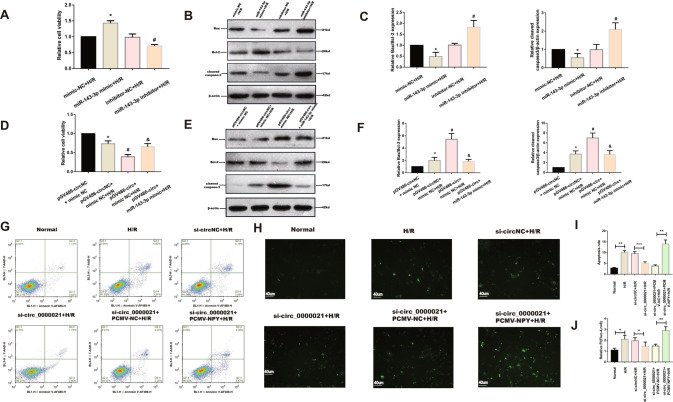
MiR-143-3p is responsible for mmu_circ_0000021-mediated calcium influx in cardiomyocytes and reduces H/R-induced cardiomyocyte apoptosis by via NPY. **A**, **D** The CCK-8 assay was used to determine relative cell viability. **B**, **E** Expression of apoptosis-associated proteins in response to injury or death, *P < 0.05 compared with the mimic-NC group+H/R, #*P* < 0.05 compared with the inhibitor-NC + H/R group; *n* = 3/group. **C**, **F** Expression of circRNA and related apoptotic proteins determined by Western blot analysis, *P < 0.05 compared with the pGV486-circNC + mimic-NC group, #*P* < 0.05 compared with the pGV486-circNC + mimic-NC + H/R group, &*P* < 0.05 compared with the pGV486-circ + mimic-NC + H/R group, *n* = 3/group. **G**, **I** Flow cytometry and quantitative analysis of the degree of apoptosis under different conditions. **H**, **J** The effects of intracellular Ca^2+^ concentrations were analyzed using fluorescence microscopy with Fluo-4 AM. *P < 0.05, **P < 0.01, vs. indicated group; *n* = 8/group.

The effects of miR-143-3p on cardiomyocyte function were next explored using loss- and gain-of-function assays. MiR-143-3p overexpression dramatically boosted cell viability following H/R, but miR-143-3p downregulation had the reverse effect (Fig. 4A). The miR-143-3p mimic also lowered calcium-induced cell death, decreased Bax and caspase 3 expression, and enhanced Bcl-2 expression. However, knockdown of miR-143-3p produced the opposite effect. (Fig. 4B, C) Next, MiR-143-3p mimic abolished the detrimental effects of mmu_circ_0000021 overexpression, suggesting that it is a downstream of mmu_circ_0000021. (Fig. 4D). Additionally, mmu_circ_0000021 overexpression mitigated the elevated expression of Bax and cleaved caspase3 and the loss in Bcl-2 following co-transfection with the miR-143-3p mimic. (Fig. 4E, F). To investigate whether apoptosis is related to changes in intracellular calcium induced by NPY, we measured calcium levels using the fluorescence probe Fluo-4am. Flow cytometry analysis showed that mmu_circ_0000021-knockdown in the H/R group attenuated apoptosis (Fig. 4G, I). Using fluorescent microscopy (Fig. 4H, J), we found that apoptosis and calcium influx were regulated by mmu_circ_0000021, providing additional evidence that mmu_circ_0000021 is a critical regulator of cardiomyocyte function. In summary, the current findings indicate that miR-143-3p has a beneficial effect on endothelial activity by disrupting mitochondrial calcium homeostasis and creating an environment conducive to apoptosis in cardiomyocytes following H/R. Our data also indicate that miR-143-3p, as a downstream target of mmu_circ_0000021, has the opposite impact on calcium overload compared to mmu_circ_0000021.

### Inhibition of mmu_circ_0000021 attenuates leukocyte infiltration in vivo

**Fig. 5:**
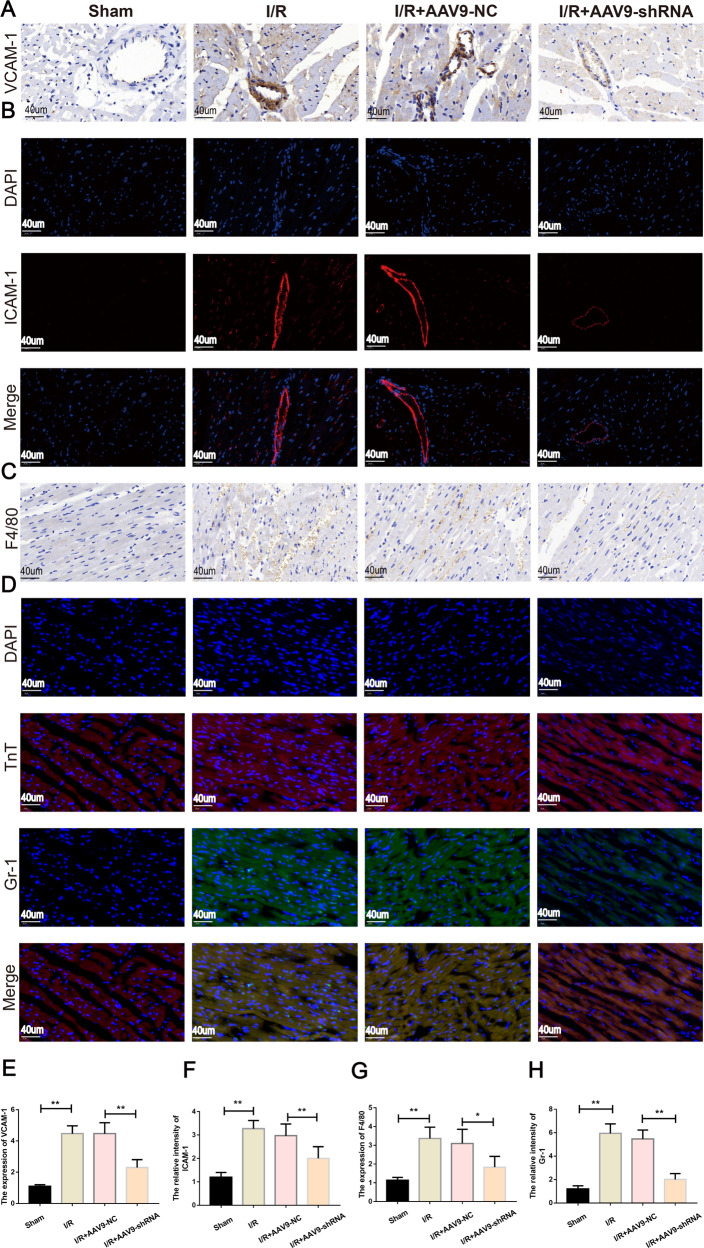
mmu_circ_0000021 suppression inhibited leukocyte infiltration in the myocardium following I/R. **A**, **E** Immunohistochemistry was used to analyze the microvasculature for VCAM-1 expression. **B**, **F** Immunofluorescence assays were used to assess ICAM-1 expression in the microvasculature. **C**, **G** Immunohistochemistry revealed the presence of neutrophils stained with F4/80. **D**, **H** Gr-1 was used to stain the neutrophils, while cTnT was used to stain the myocardium. Colocalization of Gr-1 and cTnT in the heart indicates that neutrophils have migrated into the myocardium. *P < 0.05, **P < 0.01. vs. indicated group, *n* = 8/group.

The mmu_circ_0000021 obtained from sequencing and bioinformatics prediction screening was analyzed to discover if it influences leukocyte infiltration. Leukocyte recruitment and reaction to inflamed endothelium are exacerbated by myocardial I/R, which increases the levels of VCAM1, ICAM1, and protein gamma response gene 1 (Gr-1) on the surface of microvascular endothelial cells (Fig. 5A, B, D, E, F, H). F4/80 staining was found in significant quantities following I/R in vivo (Fig. 5C, G). Compared to the I/R + AAV9-shRNA group, down-regulation of VCAM1 and ICAM1 on the microvascular surface and reduced F4/80 presence in the myocardium played a key role in myocardial I/R damage. The above results indicate that mmu_circ_0000021 knockdown protects cardiomyocytes from apoptosis and decreases leukocyte infiltration.

### Inhibition of mmu_circ_0000021 ameliorates microvascular integrity and permeability in vivo

**Fig. 6:**
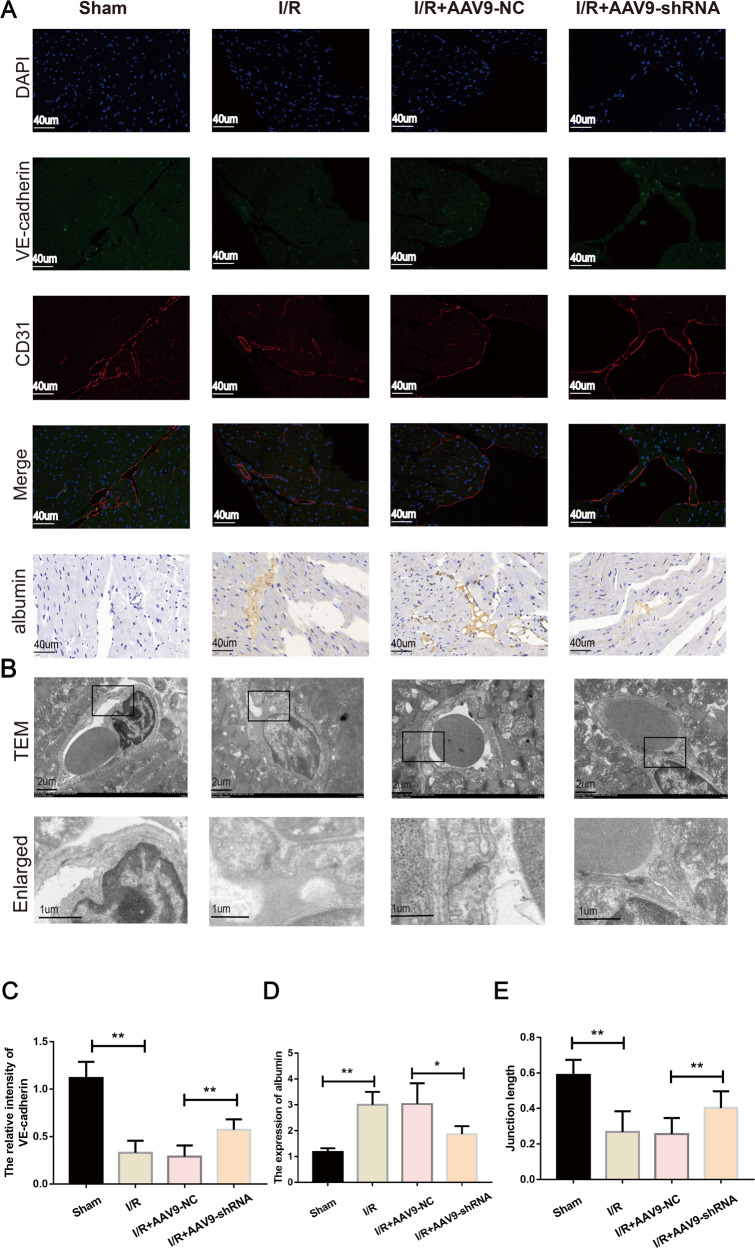
mmu_circ_0000021 suppression maintained the microvascular barrier following I/R. **A**, **C** Endothelial barrier integrity was assessed using double immunofluorescence labeling of VE-cadherin and CD31. The discontinuity in VE-cadherin expression was ameliorated by knockdown of mmu_circ_0000021. **A**, **D** Immunohistochemistry was used to determine microvascular permeability and plasma albumin levels. Absorbed plasma albumin from the microvessels during I/R was reduced by inhibiting mmu_circ_0000021. **B**, **E** Microvessel endothelial cell junction were visualized using TEM. Cellular contact and electron dense region are shown in the lower panels, which are from larger images shown in the top panels (cortical protein complex). *P < 0.05, **P < 0.01. vs. indicated group; *n* = 8/group.

Previous studies suggest that calcium ions further damage the integrity and permeability of capillaries. VE-cadherin labeling between endothelial cells was diminished following myocardial I/R (Fig. 6A), consistent with the findings presented above. We observed that electron-dense endothelial cell-cell interactions were disrupted, and the endothelium barrier integrity was constant (Fig. 6B, E) in addition to microvessel albumin leak (Fig. 6A, D). The I/R + AAV9-shRNA group showed increased microvessel surface levels of VE-cadherin and enhanced electron-dense cell connections compared to the I/R + AAV9-NC group (Fig. 6A-D). Collectively, these findings indicate that inhibiting mmu_circ_0000021 expression improves the microvascular integrity and permeability following myocardial I/R injury.

### Inhibition of mmu_circ_0000021 improves cardiac microvascular perfusion in vivo

**Fig. 7:**
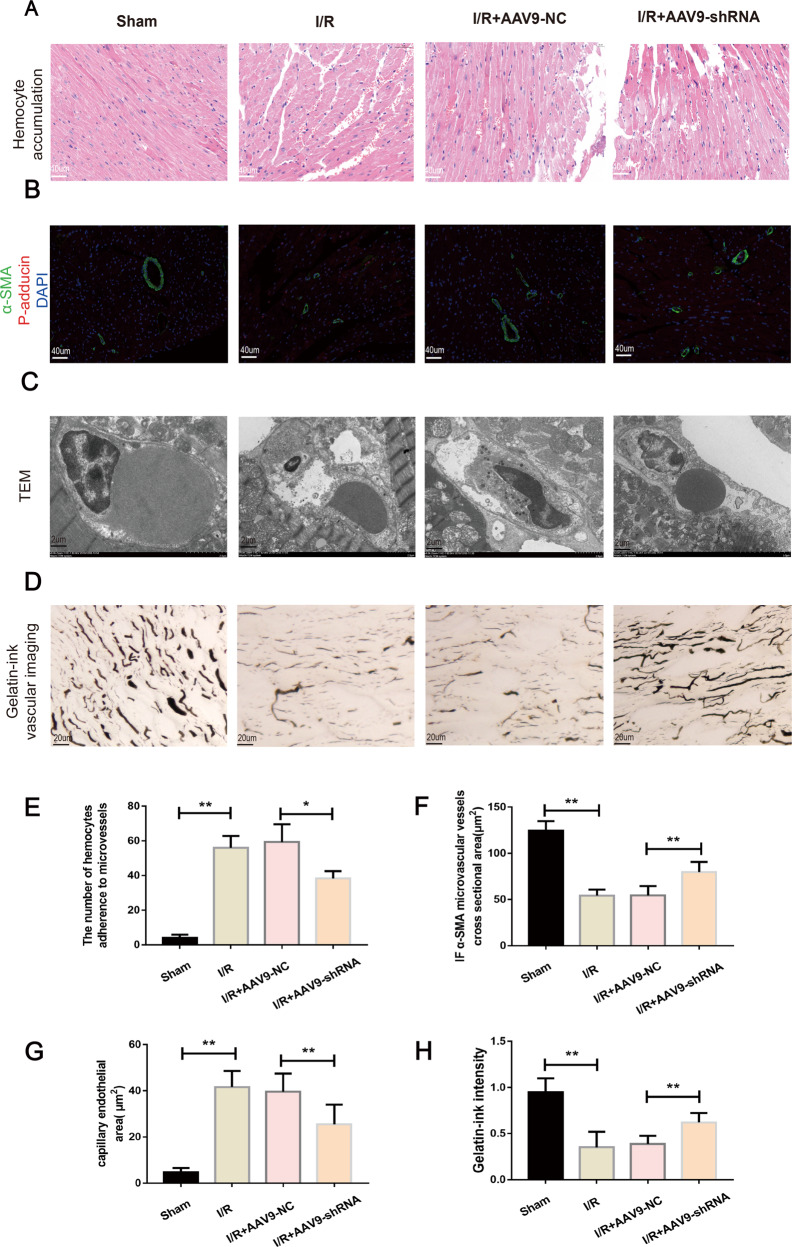
mmu_circ_0000021 suppression improved cardiac microvascular perfusion following I/R. **A**, **E** After a microvascular blockage, HE staining revealed erythrocyte aggregation and morphological alterations. **B**, **F** Immunofluorescence detection of α-SMA in microvessels. **C**, **G** Microvascular endothelial and luminal areas were assessed using TEM. mmu_circ_0000021 suppression reduced I/R-induced endothelial cell edema and hypertrophy, which resulted in luminal stenosis. **D**, **H** Microvascular perfusion was examined using gelatin-ink staining. When I/R was suppressed, mmu_circ_0000021 suppression reduced the microvessel obstruction. *P < 0.05, **P < 0.01, vs. indicated group; *n* = 8/group.

The mmu_circ_0000021 acquired by sequencing and bioinformatics prediction screening to evaluate if inhibition of circRNA expression impacts hypoperfusion of cardiac tissue. HE staining showed a change in myocardial red blood cell morphology, such that the cells swelled (Fig. 7A, E), indicating microvascular obstruction and interruption of turbulent blood flow. Myocardial I/R also induced endothelial cell swelling, leading to narrowing of the microvascular lumen. These changes across the groups were evident by immunofluorescence detection of α-SMA (Fig. 7B, F). In addition, TEM analysis showed that the microvascular lumen area was significantly reduced, and the endothelial area was increased (Fig. 7C, G). Myocardial I/R also caused microcirculation disorders, as shown by gelatin-ink staining (Fig. 7D, H). Compared with the I/R + AAV9-NC group, there was significant improvement in cell morphology, reduced endothelial cell swelling, increased microvascular lumen area, and more open microvessels in the I/R + AAV9-shRNA group (Fig. 7A-H). In summary, these observations suggest that inhibiting mmu_circ_0000021 improves cellular responses to I/R injury.

### Inhibition of mmu_circ_0000021 reduces infarct size and improves heart function in vivo

**Fig. 8:**
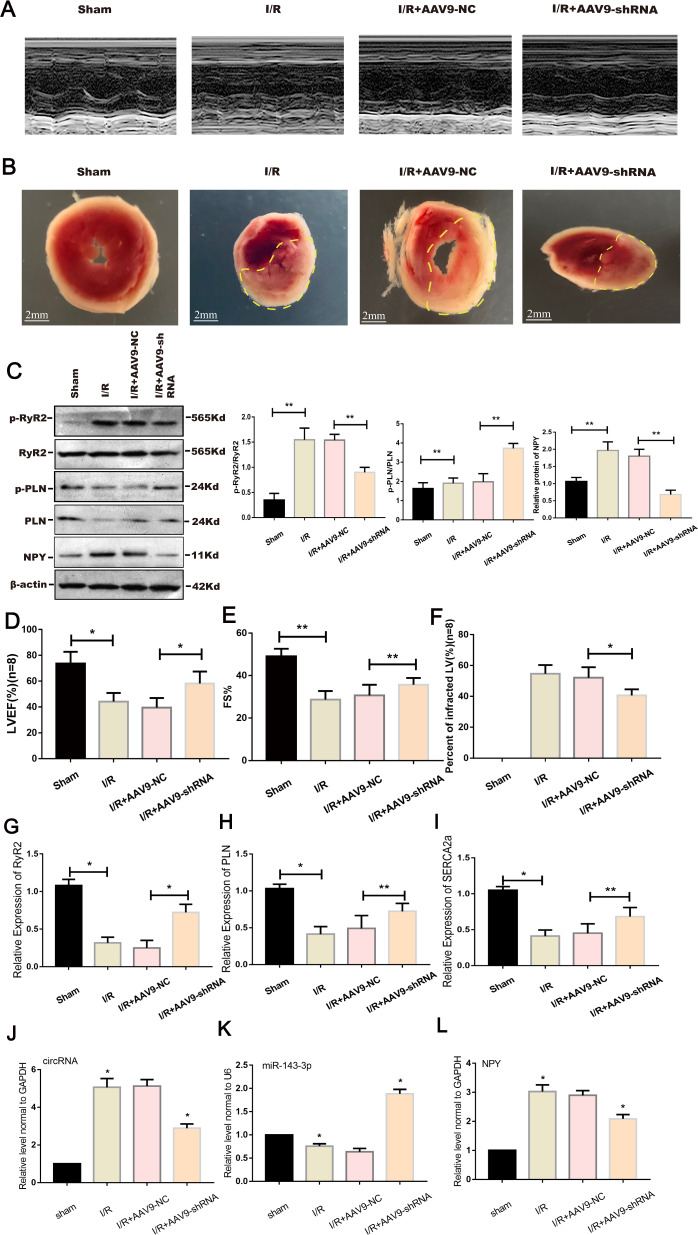
Inhibiting mmu_circ_0000021 expression reduced the infarct size and improved cardiac function following I/R. **A**, **D**, **E** LV function was determined by echocardiograms (**A**) including EF (**D**) and FS (**E**). **B**, **F** The size of the infarct and the region of the myocardial infarction were determined using TTC. **C** In the sham group, the amount of RyR2, PLN, and their phosphorylated forms were reduced. **G**–**L** qRT-PCR showed changes in PLN, RyR2, SERCA2a and the changes of miR-143-3p/NPY axis after the inhibition of mmu_circ_0000021 expression. *P < 0.05, **P < 0.01, vs. indicated group; *n* = 8/group.

We next tested if reducing circRNA expression influenced heart infarct size and cardiac function following I/R. I/R injury increased the size of the infarct (Fig. 8B, F). Furthermore, I/R damage reduced cardiac function, as demonstrated by a substantial decrease in LVEF (Fig. 8A, D, E). Thus, the pathophysiological alterations in the I/R + AAV9-shRNA group were minimized compared to the I/R + AAV9-NC group (Fig. 8A, B). Inhibiting the expression of mmu_circ_0000021 decreased infarct size and enhanced cardiac function. These findings suggest that AAV9-shRNA targeting mmu_circ_0000021 can reduce cardiac remodeling following I/R, consequently improving cardiac function in vivo. Western blot analysis revealed that the NPY, p-RyR2, and p-PLN were significantly greater in the experimental group compared to the sham group (Fig. 8C). However, at the genetic level, PLN, SERCA2a, and RyR2 declined dramatically in the sham group, indicating that a reduction in mmu_circ_0000021 might sustain their levels (Fig. 8G-I). Data on changes in the miR-143-3p/NPY axis following inhibition of mmu_circ_0000021 are presented to show the in vivo *m*echanism, which is consistent with the in vitro validation (Fig. 8J-L). Therefore, inhibiting mmu_circ_0000021 in vivo can ameliorate microcirculation and cardiomyocyte dysfunction by increasing calcium processing capacity.

## Discussion

The purpose of this work was to figure out what role mmu_circ_0000021 plays in the pathophysiology of I/R-induced. Mmu_circ_0000021 was up-regulated in the myocardium after I/R, knockdown of mmu_circ_0000021 inhibited activation of the miR-143-3p/NPY pathway, calcium influx, and apoptosis during H/R. Additionally, knockdown of mmu_circ_000021 maintained microvascular integrity and permeability, as well as restored cardiac microvascular perfusion, and successfully prevented cardiac microvascular I/R damage. CircRNA has been implicated as a factor in various cardiovascular pathologies [27]. According to a new bioinformatics study of acute MI, circUBXN7 protects cardiomyocytes from apoptosis and inflammation following H/R injury via regulating miR-622 and maintaining MCL1 expression [28]. The purpose of this study was to see if the function of miR-143-3p/NPY-mediated calcium influx response in cardiac microvascular I/R damage was a downstream mechanism of mmu_circ_0000021 [29]. Next, inhibiting mmu circ 0000021 reduces cardiac microvascular I/R damage and increases microvascular perfusion, hence enhancing microvascular integrity and reducing microvascular hyperpermeability, which promotes cardiomyocyte death and inflammation. This reduces oxidative stress, loss of cell-cell interactions, and endothelial barrier disintegration. This improves the integrity and permeability of the microvascular system while minimizing damage.

Calcium overload has long been recognized as a primary mechanism of I/R injury [30]. NPY constricts the coronary microvasculature via calcium mobilization mediated by the Y1 receptor, demonstrating the utility of blocking this receptor in lowering coronary vascular resistance and infarct size [31]. Aside from this, NPY deletion has been shown to increase cardiac function, reduce MI, and prevent cardiomyocyte death through the miR-499–FoxO4 NPY type 1 receptor-dependent pathway [32]. MI produced by persistent vascular obstruction can cause irreversible heart damage, resulting in bigger infarct areas, severe heart failure, and worse outcomes [33]. Due to the fact that coronary recanalization does not fully restore microcirculation, it worsens myocardial damage and diminishes any therapeutic advantages. These microcirculation abnormalities in cardiac tissue have the potential to exacerbate or induce subsequent damage [34]. Here, we demonstrate that miR-143-3p/NPY signaling may play a role in myocardial I/R damage, and that targeting mmu_circ_0000021 may have therapeutic value.

A single layer of epithelial cells constitutes the microvascular structure of the cardiovascular system. Because microvascular endothelial cells are exposed to circulating leukocytes, they are more susceptible to I/R damage than cardiomyocytes. The disruption of the endothelium barrier by pro-inflammatory cytokines promotes the loss of VE-cadherin-mediated cell-cell junctions. The miR-143-3p/NPY pathway, which enhances calcium influx, stimulates ICAM-1 and VCAM-1 expression in microvascular endothelial cells, hence enhancing endothelium-leukocyte adhesion. When VE-cadherin levels in endothelial cells fall, leukocyte adhesion, aggregation, and inflammatory cytokines rise. We were able to observe these cell connections and ascertain the integrity of the barrier. Calcium overload and inflammatory activation both contribute to endothelial cell death and reduced endothelial viability, both of which result in microvascular damage. Consequently, albumin in the microvascular lumen might impact heart tissue. These pathophysiological processes can lead to microvascular I/R degradation and microcirculatory perfusion deficiencies (Supplementary Fig. 1 shows how circular RNA affects the distribution of Ca^2+^ through the miR-143-3p-NPY axis).

A limitation of the current study should be noted. While the animal studies described here focused on the importance of low mmu_circ_0000021 expression in retaining microvascular function and NMCMs were also used in vitro. However, it is not an endothelial cell line. As a result, we will use endothelial cell lines to examine the function of mmu_circ_0000021 in the future.

In conclusion, we explored the role of the circRNA-miRNA-mRNA axis and mmu_circ_0000021 was related to the distribution of Ca^2+^ in NMCMs and further exerted the positive functions in the initiation, development of microvascular disorder. Future treatment should focus on preventing microcirculation reperfusion injury. CircRNA is now being studied as a molecular-targeted therapy for microvascular reperfusion injury. This study identifies molecular pathways and new therapeutic targets in microvascular function.

## Material and methods

### Materials

Antibodies for anti-phospholamban (PLN), anti-RyR2, anti-p-PLN, anti-p-RyR2 were from Abcam (Cambridge, UK). Abcam (Britain) also provided the α-SMA and p-adducin proteins. Cell Signaling Technology provided NPY protein (Product Number: 11976). Other antibodies were as follows: F4/80 (1:100, Bioss, bs-11182R), vascular endothelin-cadherin (1:100, Bioss, bs-0878R), CD31 (1:100, Abcam, ab24590), intracellular adhesion molecule-1 (ICAM-1) (1:500, Abcam, ab171123), vascular cell adhesion molecule-1 (VCAM-1) (1:500, Abcam, ab134047), cardiac troponin (cTnT) (1:400, Abcam, ab45932), and albumin (1:1,000, Abcam, ab8940). Beyotime Biotechnology (China, Product Number: S1060) provided Fluo-4/AM. Thermo Fisher Scientific and Takara Bio provided the reverse transcription (RT) kit and the ABI Prism 7500 Real-Time PCR equipment with the SYBR® RT-PCR kit. Takara designed and synthesized the primers. Hanbio Inc. (Shanghai, China) developed and manufactured the miR-143-3p mimic, miR-143-3p inhibitor, mimic negative control (NC), inhibitor NC, small interfering RNA (siRNA), and siRNA-NC. Hanbio Inc. (Shanghai, China) created an adeno-associated virus (AAV) with a short hairpin RNA (shRNA) targeting the mmu_circ_0000021 sequence (AAV-shRNA) and a negative control AAV (AAV-NC).

### Animals

Junke Animal Co., Ltd. provided healthy adult male C57BL/6 mice weighing 18–22 g (Nanjing). The C57/B6J mice were bred and maintained in a standard environment (23 ± 1 °C and 55 ± 5% humidity). Prior to all experiments, the mice were given unlimited access to water and food and kept on a light/dark cycle for at least one week.

### Animal model establishment

Pentobarbital sodium (50 mg/kg) was injected into the abdominal cavity to anesthetize the mice. To create the myocardium I/R model, the pericardium was opened to expose the heart, and the mouse was treated with 45 min of regional ischemia followed by 180 min of reperfusion [35, 36]. In a total volume of 25 L, mice received intramyocardial injections of 1 × 10^12^ vp/ml AAV9 circ_0000021 (*n* = 8), AAV9-NC (*n* = 8), or saline (*n* = 8) at four distinct locations in the peri-infarct region (basal anterior, mid anterior, apical anterior, and apical lateral). Echocardiography was used to monitor left ventricular (LV) functional changes at 1, 2, 3, and 4 weeks after myocardial infarction (MI), as well as structural remodeling at 3 weeks [37]. Eight-week-old male C57BL/6 mice weighing 18–22 g were randomly divided into four groups (*n* = 8 mice per group): (1) Sham group: sham mice underwent left thoracotomy without left anterior descending arterial (LAD) ligation or injection; (2) I/R model group: mice underwent left thoracotomy with LAD ligation but were injected with saline (100 ul); (3) I/R + AAV9-NC group: mice underwent the procedure but were administered control shRNA (4) I/R + AAV9-shRNA group: animals were given the same treatment as the I/R group but were also given AAV9-shRNA (Supplementary Fig. 2A illustrates the procedure).

### Cell culture and H/R model establishment

Neonatal mouse ventricular myocytes from 1-day-old to 3-day-old C57BL/6 mice were isolated under aseptic procedures. Ophthalmic scissors were used to repeatedly cut the heart ventricles into 1 - 2 mm^3^ fragments. Using 0.05% collagenase type II (Sigma), the tissues were chopped and digested by trituration eight to ten times for 5 minutes each time. DMEM/F12 (1:1) with 20% fetal bovine serum (Invitrogen) was used to resuspend the cells after discarding the supernatant. To mimic I/R in vitro, the cells were placed in a 37 °C hypoxic incubator with 5% CO_2_ + 95% N_2_ for 6 hours and then reoxygenated with 95% air + 5% CO_2_ for 6 hours (Supplementary Fig. 2B) [38].

### Screening of differentially expressed circRNAs and bioinformatics prediction

Genes were screened from the mouse cardiac I/R model using high-throughput circRNA sequencing. For the Sham (*n* = 4) and I/R (*n* = 4) groups a *P* value 0.05 and fold change > 2 or 0.5 was chosen as the criterion for identifying differentially expressed circRNAs. To evaluate circRNA/miRNA/mRNA interactions, R analysis software was used to map the differentially expressed genes into a cluster analysis graph, and bioinformatics tools including TargetScan (TargetScan.org/vert 71/) [39], Miranda (Microrna.org/microrna/Home.Do), and circBase (http://www.circbase.org/) [40] were used to analyze the clusters. Venn Diagram 2.1.0 (https://bioinfogp.cnb.csic.es/tools/venny/) was utilized to further investigate overlapping differentially expressed genes between the two data sets [41].

### Gene Ontology (GO) and Kyoto Encyclopedia Genes and Genomes (KEGG) analyses

GO analysis is a well-accepted method for gene annotation [42]. The molecular interactions and relation networks for metabolism were annotated using KEGG (genome.jp/kegg/) in a collection of manually drawn pathway maps [43]. Differentially expressed circRNA‐associated genes were based on network scores or a -log10 (*P*-value) representing the significance of enriched focal genes.

### Echocardiography, transmission electron microscopy (TEM), triphenyltetrazolium hydrochloride (TTC) staining, and gelatin-ink staining

The LV ejection fraction (LVEF) was measured using a Philips Sonos7500 ultrasound system (Philips Medical, Amsterdam, Netherlands). The microvascular ultrastructure of the cardiac I/R model was examined using TEM (HT7800, Tokyo, Japan). We used Image J 1.48 (National Institutes of Health) to measure the capillary lumen area and endothelial cell area (NIH, Bethesda, MD, USA) [44]. TTC hydrochloride (Sigma-Aldrich, USA) was applied to the heart tissue for 10 min at 37 °C, and then the heart was fixed in 10% neutral buffered formaldehyde for 24 h before further processing. Using a Leica microscope, the tissue slices were imaged and then quantified using Image J. Paraformaldehyde-fixed and paraffin-embedded heart tissues were sectioned at 6–7 mm. India ink (2%) consisting of 20% gelatin and normal saline was injected into the heart with a syringe until the heart was filled with ink. With a 40x multiplier objective, the slices were analyzed using a DMR + Q550 automatic image analyzer (Leica, Germany).

### Histopathology, immunofluorescence, and immunohistochemistry

The mouse myocardium was fixed in 4% paraformaldehyde, dried in an ethanol gradient, and then transferred to a xylene solution before being embedded in paraffin. Hematoxylin and eosin (HE) stained sections (4 um thick) were analyzed to demonstrate inflammatory cell infiltration and microvascular alterations. The tissue sections were also stained with immunofluorescent antibodies using a standard staining method. Each section was examined in five high-magnification fields (40 x), and the high-density area of each section in each group was randomly rated.

### miRNA, plasmid transfection, and RNA interference

An miR-143-3p mimic and inhibitor were transfected into the cardiomyocytes. Transfection media was replaced 6 hours after the start of transfection, and newborn mouse cardiomyocytes were harvested 48 hours after the start of transfection for further study. The miR-143-3p mimics (miR-143-3p mimic), miR-143-3p inhibitors (miR-143-3p inhibitor), NC mimics (miRNA-NC), and NC inhibitors (inhibitor-NC) were synthesized by Shanghai Hanbio Co, Ltd. Similarly, we transfected AAV-9 virus into the neonatal mouse cardiomyocytes according to the Hanbio adenovirus operating manual. The synthetic vector was transfected into the cardiomyocytes using Liposome 3000 (Invitrogen, USA). Cardiomyocytes were also transfected with an appropriate negative control per the manufacturer’s instructions.

### Measurements in Ga^2+^ cardiomyocytes

Fluo-4/Am (6 mol/L) was incubated with the Ca^2+^-loaded cardiomyocytes for 30 minutes at 37 °C before the excess Fluo-4 AM was removed. The neonatal mouse cardiomyocytes (NMCM) loaded with Ca^2+^ were subjected to H/R or the other intervention conditions during imaging with a confocal microscope to measure calcium transients. The duration and spread of the calcium transients were recorded as full duration at half maximum (FDHM) [45, 46].

### Cell viability assay

Cell viability was assessed using the Cell Counting Kit-8 (CCK8) assay (Biosharp). The CCK8 reagent was added to each well of a 96-well plate in accordance with the manufacturer’s instructions and incubated at 37 °C for 2 hours. Infinite M200 was used to compute the optical density at OD450 (Tecan, Switzerland).

### RNA isolation and qRT-PCR

Total RNA was extracted from cardiac tissues and cells using Trizol (Invitgen, USA). RNA quality and amount were determined using a Nanodrop (NanoDrop Technologies; Thermo Fisher Scientific). The total RNA concentration in the samples was determined using qRT-PCR (TaKaRa, Dalian, China). For non-coding RNA and mRNA, the internal reference was α-actin or U6. The ABI 7500 Real-Time PCR equipment was used with a total volume of 20 ul, including 10 ul of SYBR green PCR 2 master mix. The quantitative PCR settings were 95 °C for 10 min, 95 °C for 15 s, 60 °C for 0.5 mins, and 72 °C for 0.5 min, for a total of 40 cycles. To normalize the mean expression levels of internal reference genes, the 2-CT technique was employed. Supplementary Table 1 shows the primer sequences utilized, and all experiments were performed three times.

### Western blot analysis

Cultured cardiomyocytes were lysed in buffer from Roche Applied Sciences, and the cell samples and heart tissues were homogenized in RIPA lysis buffer (pH 8.0, 150 mmol/L NaCl, 50 mmol/L Tris-HCl, 1% NP-40, and 0.1% SDS). A BCA kit was used to measure protein concentration (BCA Protein Assay Kit, P0010). A 10% SDS-PAGE (Bio-Rad) was used to separate proteins, which were then transferred to a polyvinylidene fluoride membrane (0.45-m, micropore) and blocked with 5% bovine serum albumin at 25 °C for 60 min. The samples were incubated with primary antibodies for 24 h at 4 °C followed by incubation with secondary anti-rabbit/mouse IgG antibody for 60 min at 25 °C. A substrate kit (TERMO) was utilized to assess the level of immunoreactivity.

### Dual-luciferase reporter assay

The complete binding area of mmu_circ_0000021 was introduced into the pcDNA3.1 vector after amplification, and the luciferase gene was placed downstream. Targeted mutations were carried out based on the predicted binding sites of miRNA and circRNA. To measure transfection effectiveness, the Renilla luciferase plasmid (Japan Takara) with pcDNA3.1 vector was used as a reference control. Cardiomyocytes were co-transfected with the miR-143-3p mimic, mmu_circ_0000021 wildtype/mutant, and luciferase reporter vector. The cells were lysed after 36 h of treatment, and the luciferase activity level was evaluated using a dual-luciferase reporter analysis system. We compared the activity of firefly luciferase to that of Renilla luciferase. The experiment was carried out three times.

### RNA pull-down assay

Using Biotin RNA Labeling Mix (Roche Diagnostics), miR-143-3p mimics and negative controls were biotinylated and subsequently transfected into cardiomyocytes. The RNA was extracted using the RNeasy Mini kit after the cells were collected, washed, lysed, and treated with streptavidin-coated magnetic beads for 3 hours at 4 degrees Celsius (Qiagen). Finally, the abundance of mmu_circ_0000021 was measured by qPCR.

### RNA immunoprecipitation (RIP)

The RIP assay was carried out by using a Magna RIP RNA Binding Protein Immunoprecipitation Kit (Millipore) according to the manufacturer’s instructions. The antibodies against AGO2 and IgG used for the RIP assays were purchased from Abcam (ab5072, Cambridge, MA, USA).

### Flow cytometry

Cardiomyocyte apoptosis was measured using flow cytometry. After harvesting, the cells were resuspended in a phosphate buffered solution. Neonatal mouse cardiomyocytes were stained with Annexin V/FITC and PI solution, according to the manufacturer’s instructions (Annexin V-FITC Apoptosis Staining Kit, Abcam). The FACS Calibur Flow Cytometer was used to identify apoptotic cells, which were then analyzed using Flow Jo 10.0 software.

### Data analysis

The mean and standard deviation (SD) are shown. Repeated measures one-way ANOVA and Tukey’s honest significant difference tests were used to compare groups. A *P* value of <0.05 was considered statistically significant in all analyses as determined using GraphPad Prism 8.

## Supplementary information

original western blots

Supplemental Information

## Data Availability

All data generated or analyzed during this study are included in this published article.
