# Supplementary material for: CircRNA mmu_circ_0000021 regulates microvascular function via the miR-143-3p/NPY axis and intracellular calcium following ischemia/reperfusion injury
Source: Cell Death Discov. 2022 Jul 11;8:315. doi: 10.1038/s41420-022-01108-z (PMC9276824; doi:10.1038/s41420-022-01108-z)
Supplement: Supplementary file 1 — original western blots [file 41420_2022_1108_MOESM1_ESM.pptx]

## Slide 1
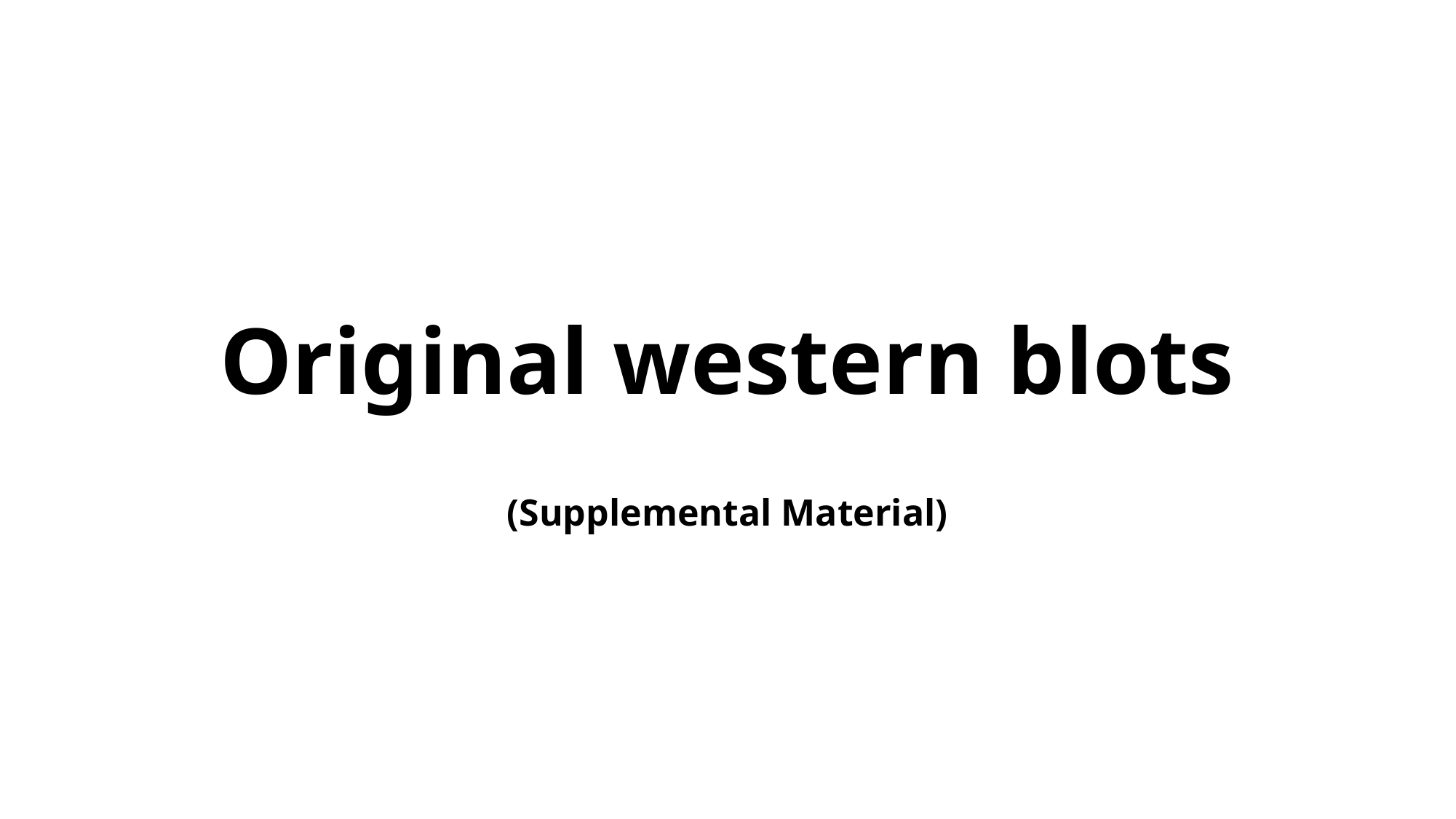

# Original western blots
(Supplemental Material)

## Slide 2
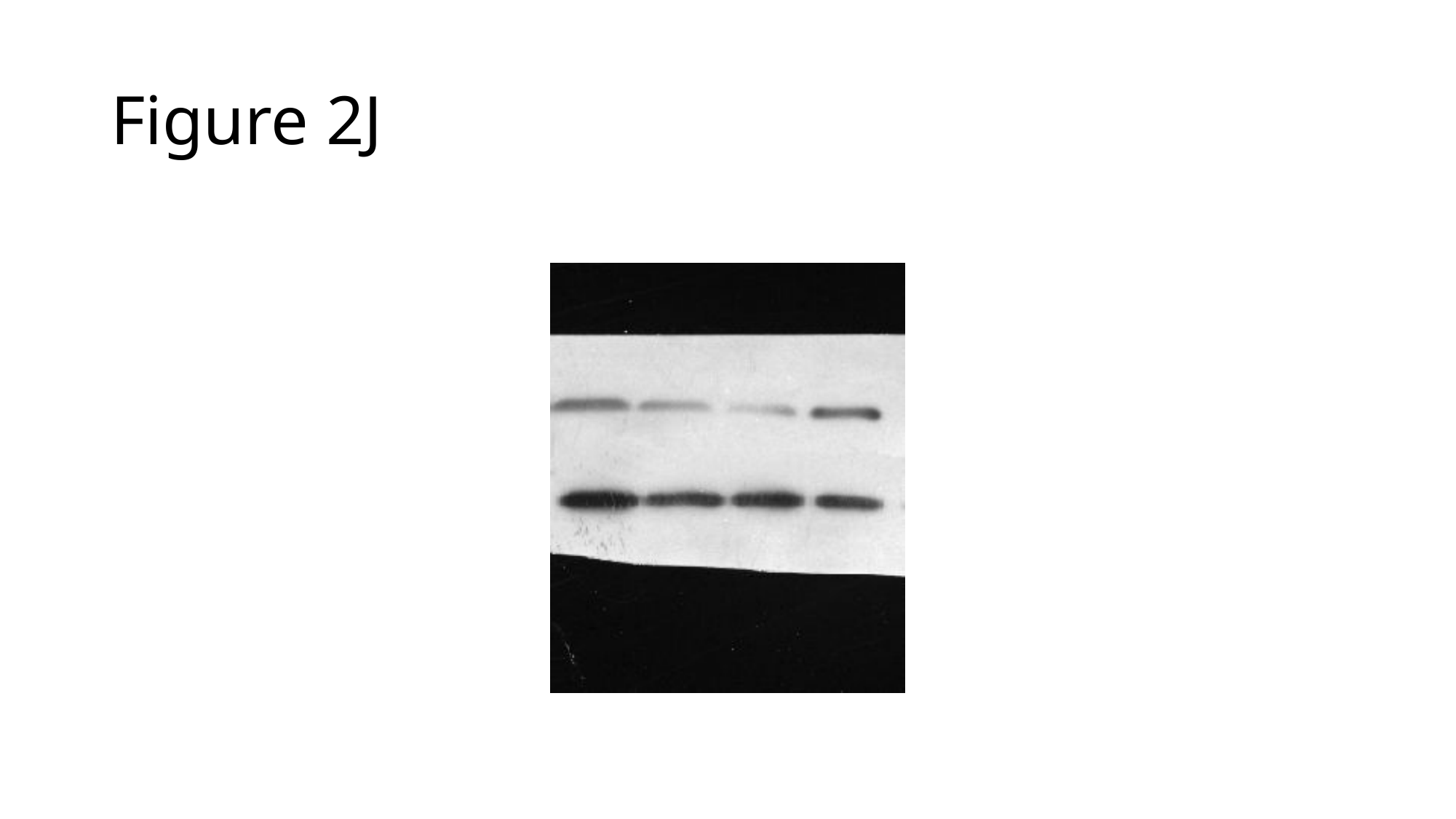

# Figure 2J

## Slide 3
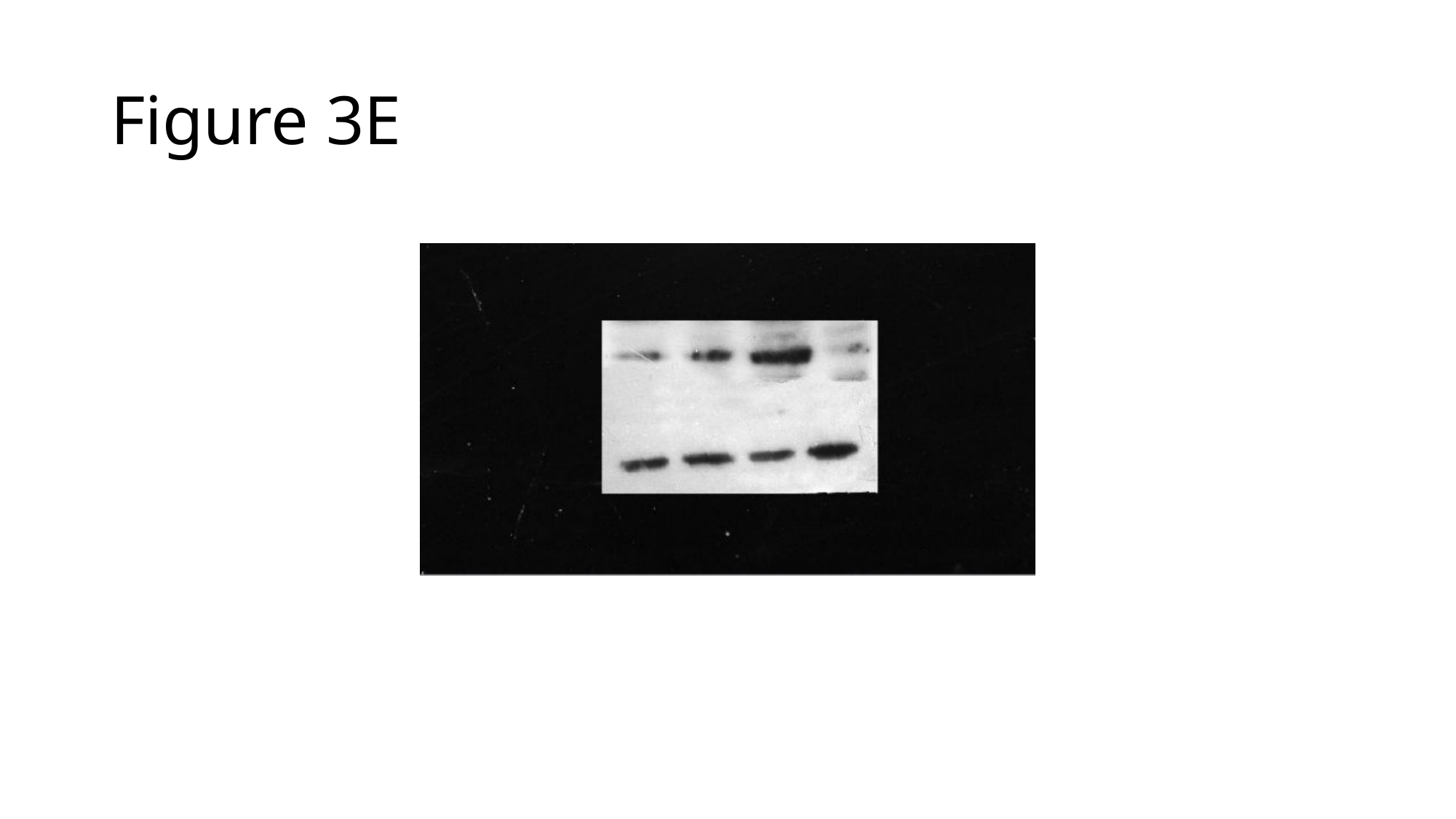

# Figure 3E

## Slide 4
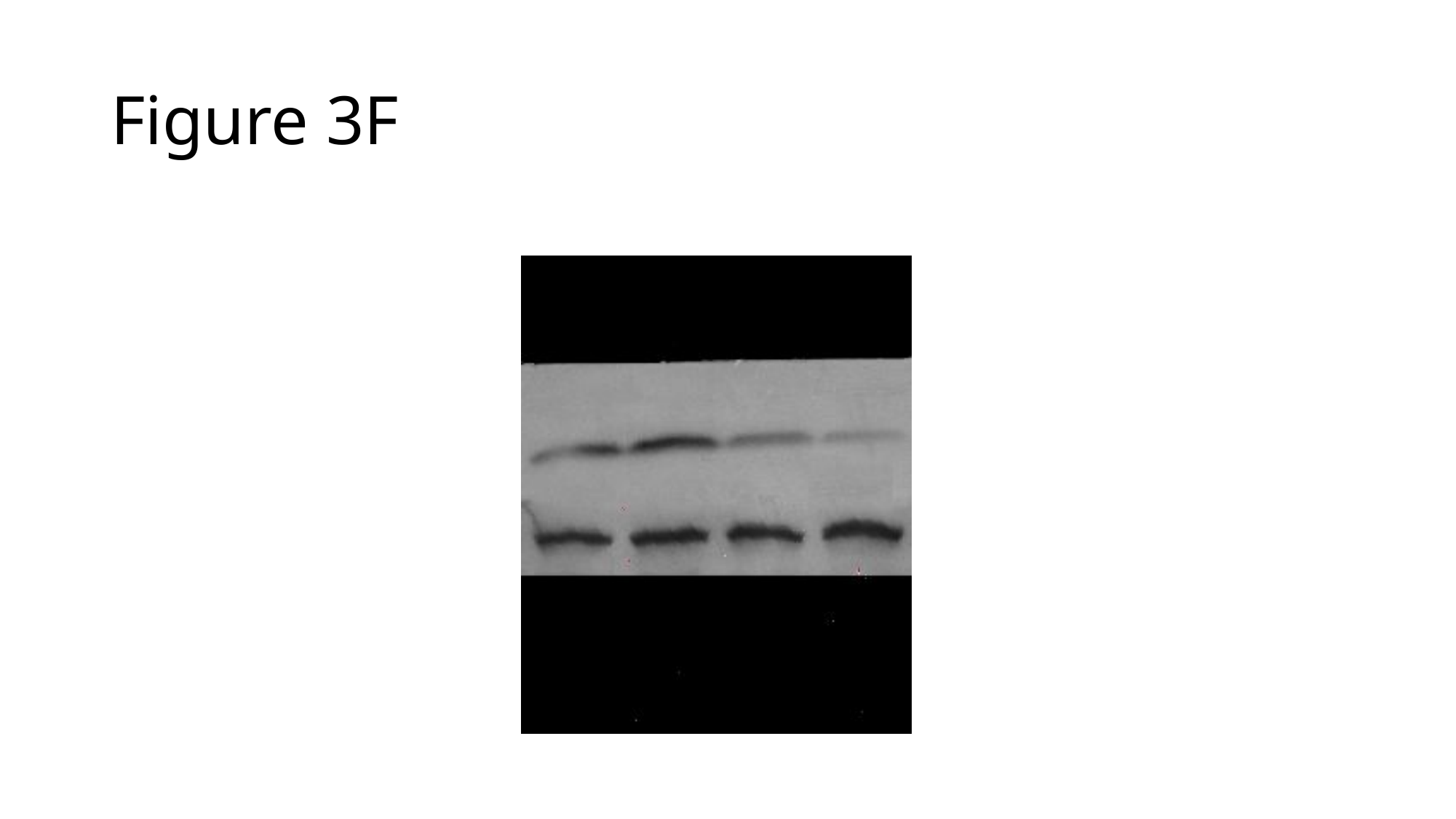

# Figure 3F

## Slide 5
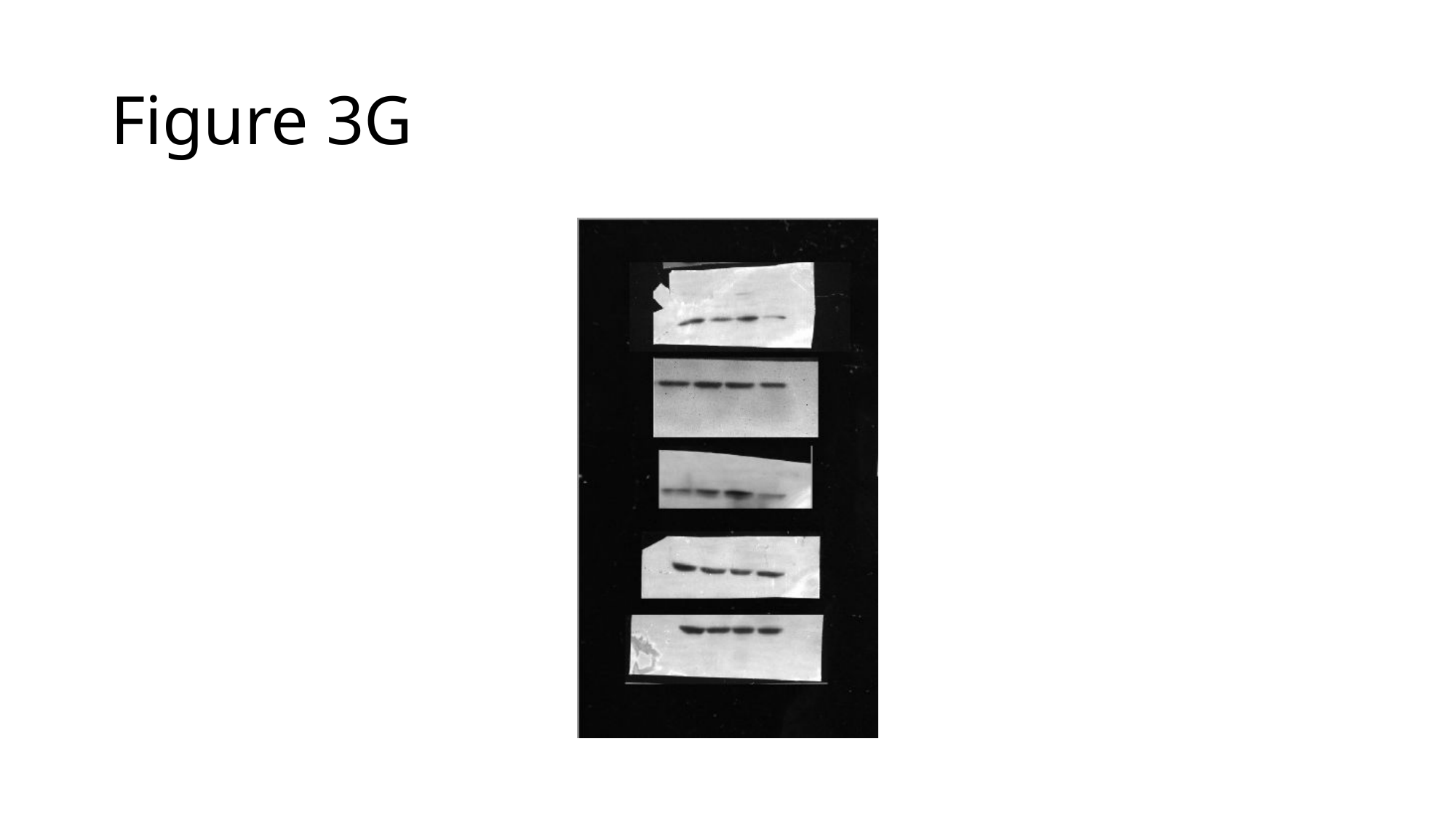

# Figure 3G

## Slide 6
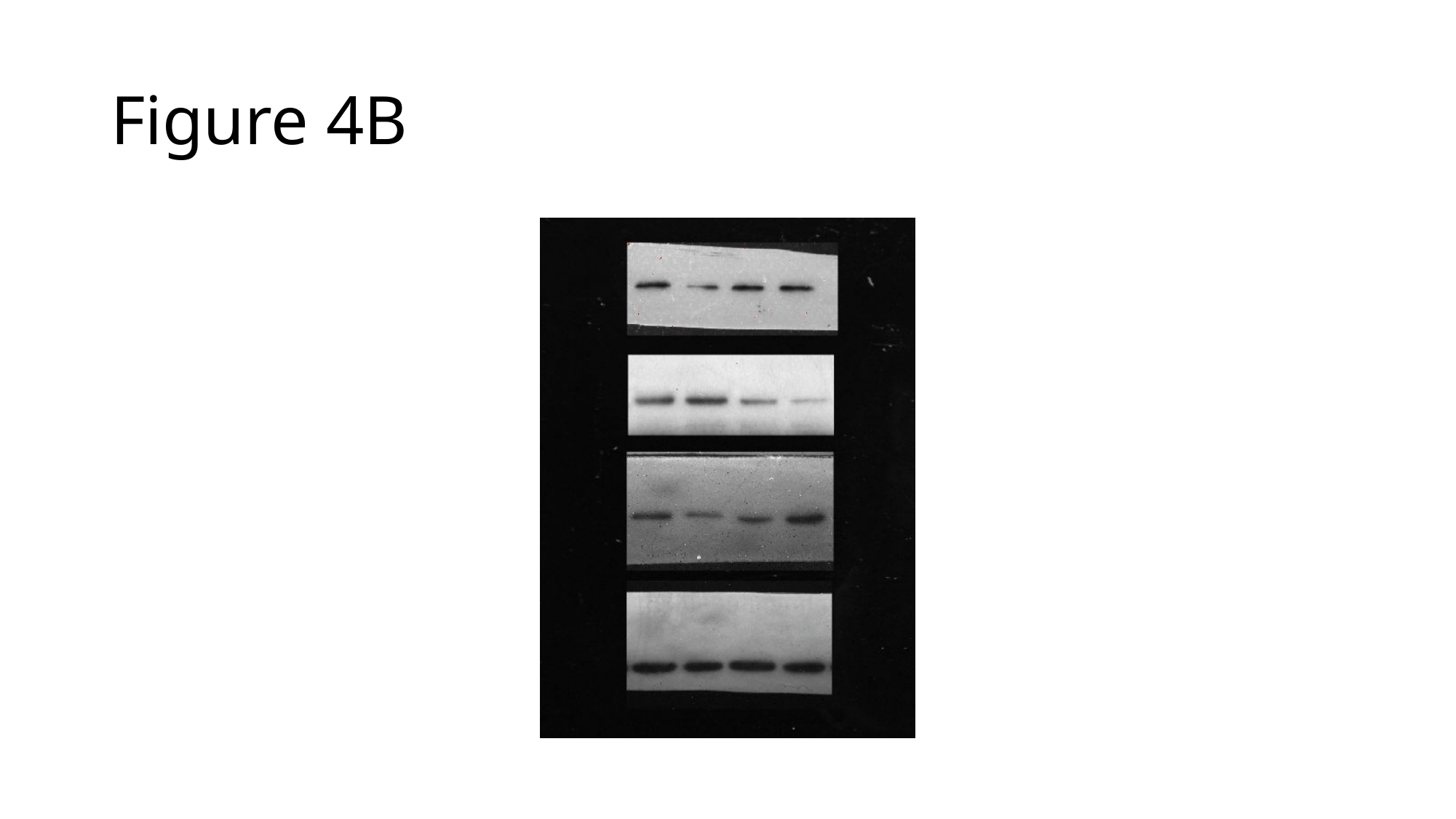

# Figure 4B

## Slide 7
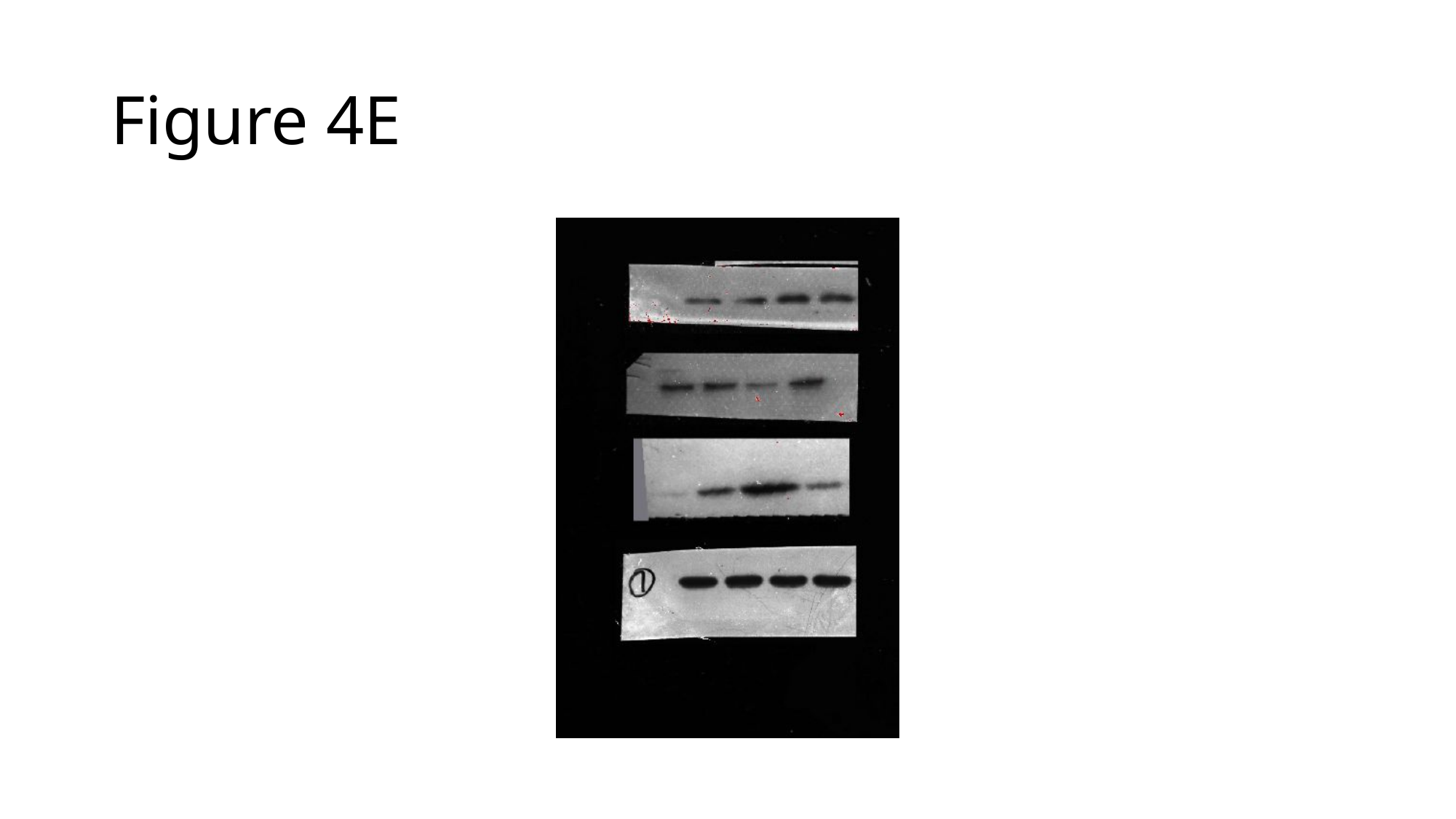

# Figure 4E

## Slide 8
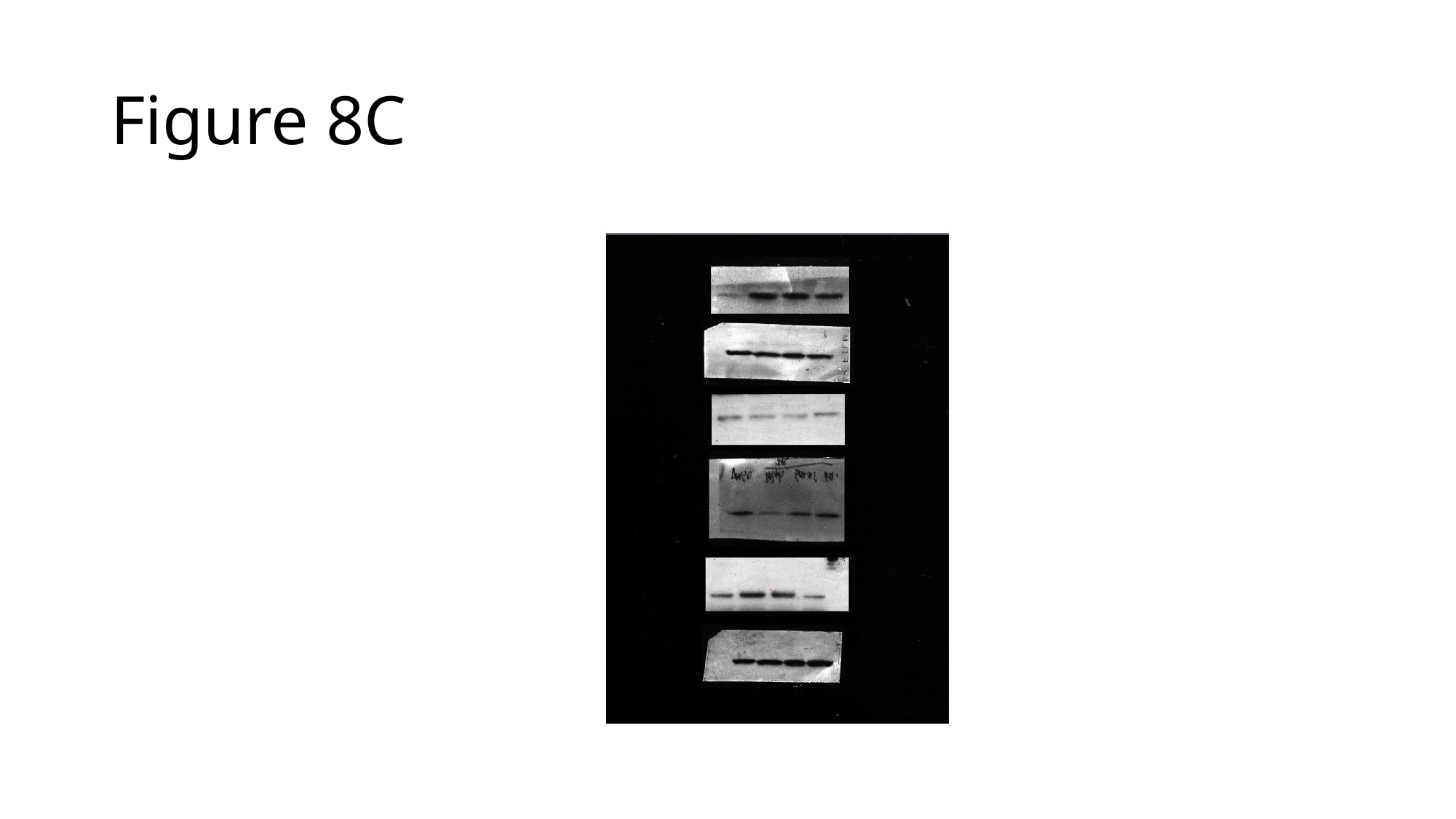

# Figure 8C
