# Supplementary material for: CircRNA mmu_circ_0000021 regulates microvascular function via the miR-143-3p/NPY axis and intracellular calcium following ischemia/reperfusion injury
Source: Cell Death Discov. 2022 Jul 11;8:315. doi: 10.1038/s41420-022-01108-z (PMC9276824; doi:10.1038/s41420-022-01108-z)
Supplement: Supplementary file 2 — Supplemental Information [file 41420_2022_1108_MOESM2_ESM.pdf]

Supplemental Table 1. RT-qPCR primers

Target genes	Forward primers	Reverse primers
NPY	5’-CGCTCTGCGACACTACATCAAT-3’	5’-TGAGATGAGGGTGGAAACTTGG-3’
miR-143-3p	5′-TGAGATGAAGCACTGTAGCTC-3′	5′-GCTACAGTGCTTCATCTCATT-3′
mmu_circ_0000021	5’-CTGCCTGTCTATCATCTCATCCAA-3’	5’-CATCGGTAAGCACAGTTTTACATCC-3’
mmu_circ_0001098	5’-GGAGAGACTGGAAGGCTGGT-3’	5’-TTTCGATGTGGTTCGTGGAG-3’
RyR2	5’-TTGTTTCCTTTTTAGAAAATGACTTC-3’	5’-GGGTGTGCAGATGTACATGC-3’
PLN	5’-TTTACAAGATCCAGCCGATGAT-3’	5’-CTGCTGATCTGCATCATTGTGA-3’
SERCA2a	5’-GGTTTTCAGTGGCTGAGGAA-3’	5’-TCCTGAGAATCACTGCTCCC-3’
β-actin	5’-CGACCACACACAGAAGGAGAT-3’	5’-GCCGATTCACACCGAGTA-3’
U6	5'-CAAATTCGTGAAGCGTT -3’	5' -TGGTGTCGTGGAGTCG-3’

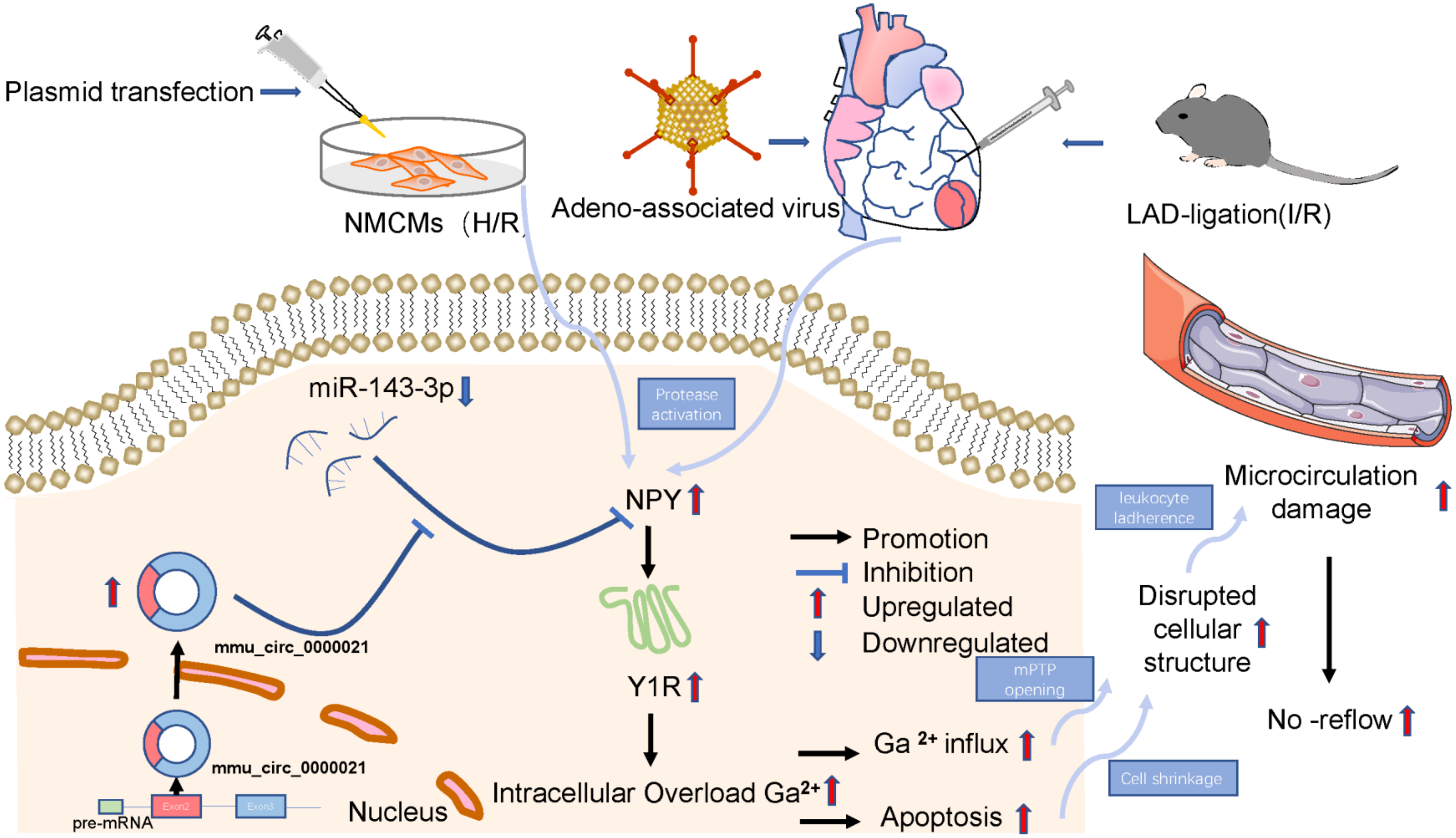

Supplemental Figure 1. **Diagram showing how circRNA affects the distribution of Ca^{2+} through the miR-143-3p-NPY axis, leading to microvascular dysfunction.**

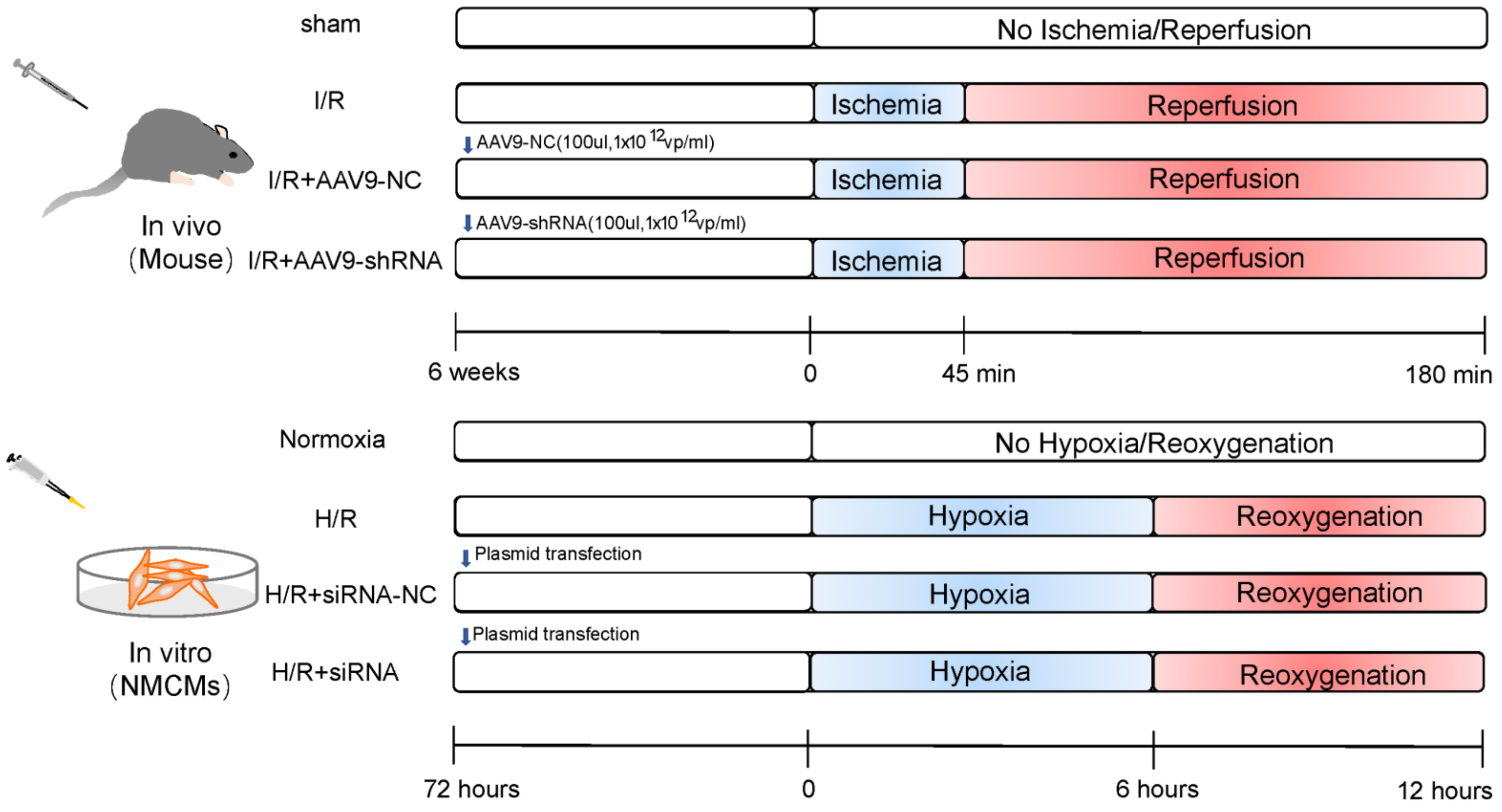

Supplemental Figure 2. **Schematic for experimental protocols. (A)** Serotype 9 AAV vectors (AAV9) expressing shNC or shRNA (AAV9-NC and AAV9–shRNA) were injected directly into the ventricle. Mice were given 45 minutes of ischemia and 3 hours of reperfusion three days later. **(B)** NMCMs were transfected with the plasmid. To induce H/R damage, NMCMs were made hypoxic for 6 hours and then reoxygenated for 6 hours.
